# Nucleoside Reverse Transcriptase Inhibitor Exposure Is Associated with Lower Alzheimer’s Disease Risk: A Retrospective Cohort Proof-of-Concept Study

**DOI:** 10.3390/ph17040408

**Published:** 2024-03-22

**Authors:** Tiffany W. Chow, Mark Raupp, Matthew W. Reynolds, Siying Li, Gwendolyn E. Kaeser, Jerold Chun

**Affiliations:** 1 IQVIA, Durham, NC 27703, USA; tiffany.chow@pennmedicine.upenn.edu (T.W.C.); mark.raupp@iqvia.com (M.R.);; 2 Perelman School of Medicine, University of Pennsylvania, Philadelphia, PA 19104, USA; 3 Center for Genetic Disorders and Aging Research, Sanford Burnham Prebys Medical Discovery Institute, La Jolla, CA 92037, USA

**Keywords:** Alzheimer’s disease, human immunodeficiency virus, nucleoside reverse transcriptase inhibitor, protease inhibitor, reverse transcriptase, reverse transcriptase inhibitor, somatic gene recombination

## Abstract

Brain somatic gene recombination (SGR) and the endogenous reverse transcriptases (RTs) that produce it have been implicated in the etiology of Alzheimer’s disease (AD), suggesting RT inhibitors as novel prophylactics or therapeutics. This retrospective, proof-of-concept study evaluated the incidence of AD in people with human immunodeficiency virus (HIV) with or without exposure to nucleoside RT inhibitors (NRTIs) using de-identified medical claims data. Eligible participants were aged ≥60 years, without pre-existing AD diagnoses, and pursued medical services in the United States from October 2015 to September 2016. Cohorts 1 (N = 46,218) and 2 (N = 32,923) had HIV. Cohort 1 had prescription claims for at least one NRTI within the exposure period; Cohort 2 did not. Cohort 3 (N = 150,819) had medical claims for the common cold without evidence of HIV or antiretroviral therapy. The cumulative incidence of new AD cases over the ensuing 2.75-year observation period was lowest in patients with NRTI exposure and highest in controls. Age- and sex-adjusted hazard ratios showed a significantly decreased risk for AD in Cohort 1 compared with Cohorts 2 (HR 0.88, *p* < 0.05) and 3 (HR 0.84, *p* < 0.05). Sub-grouping identified a decreased AD risk in patients with NRTI exposure but without protease inhibitor (PI) exposure. Prospective clinical trials and the development of next-generation agents targeting brain RTs are warranted.

## 1. Introduction

Alzheimer’s disease (AD) is the most common form of dementia, affecting an estimated 6.5 million Americans including more than 10% of Americans over 65 years of age [1]. There are no therapies that demonstrably stop the disease [2] despite hundreds of clinical trials. The recent identification of reverse transcriptase (RT)-mediated SGR in the human brain [3] (graphical abstract), which becomes dysregulated in sporadic AD, implicates FDA-approved reverse transcriptase inhibitors (RTIs) as potential therapeutics for AD [4,5].

SGR affects the amyloid-β precursor protein (*APP*) gene [3,6,7,8]. The APP protein is cleaved to produce Aβ, which comprises the senile plaques that are a hallmark of AD. Germline *APP* single-nucleotide variations and copy number variations are considered causal in rare familial AD [9,10], consistent with *APP* trisomy producing AD neuropathology in Down syndrome (DS) [11]; however, *APP* germline alterations are absent in sporadic AD, which is the most common form [1,12,13]. However, somatic and mosaic increases in *APP* copy number were identified in sporadic AD neurons as thousands of previously unrecognized variant *APP* gene and RNA sequences [3,6,14], including some containing known disease-causing familial AD variations that were absent from non-diseased brains. These *APP* variants have been identified in the brains of people with DS [7] and independently confirmed in the brain [6] and blood plasma [8] of people with sporadic AD. In vitro experiments identified three mechanistic requirements for SGR: *APP* transcription, DNA strand breaks, and RT activity [3].

The reliance of SGR on RT activity suggested that RTIs might be useful in preventing and/or treating AD [3,4,5]. Multiple FDA-approved RTIs, the first of which was approved in 1987, are currently used to treat human immunodeficiency virus (HIV) and hepatitis B [15,16]. RTIs can be orthosteric (bind to the active site) nucleoside RTIs (NRTIs) or allosteric non-NRTIs (NNRTIs), and together with integrase inhibitors and PIs, represent the components of combined antiretroviral therapy (cART) [16]. Because of effective cART, tens of thousands of people with HIV have lived to older ages [17] but are now at risk for AD, providing an opportunity to retrospectively examine the incidence of AD by assessing medical claims databases. Preliminary support for a possible effect appeared in the first peer-reviewed report of a patient with HIV and sporadic AD in 2016 [18], which contrasted with over a decade of expectations for vastly increased AD incidence in people with HIV [19,20]. Here we report results from a large, real-world dataset for the incidence of AD in aging people with HIV receiving distinct elements of cART.

## 2. Results

### 2.1. Study Participant Characteristics

**Figure 1:**
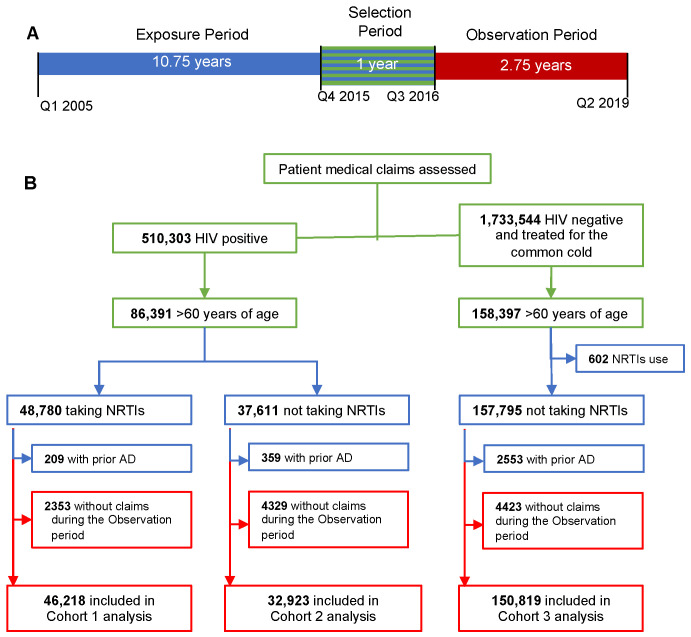
Study design and inclusion/exclusion criteria. (**A**) Timeline for the study. Patients were identified during the selection period from Q4 2015 to Q3 2016 (green). Exposure to combined antiretroviral therapies (cARTs) was determined from Q1 2005 to Q3 2016 (blue). Alzheimer’s disease (AD) incidence was determined during the 2.75-year observation period from Q3 2016 to Q2 2019 (red). (**B**) Selection of patients based upon inclusion and exclusion criteria; timing of criteria use is noted by color.

Our criteria identified 510,303 people with HIV, of whom 86,391 were aged 60 years or older in 2015 (Figure 1); 46,218 were included in Cohort 1 (HIV+/NRTI+), and 32,923 were included in Cohort 2 (HIV+/NRTI−). There were 150,819 controls in Cohort 3. The “reported alive” statistic was used to estimate mortality by an absence of insurance claims during the observation period [21]. The number of patients censored by the “reported alive” methodology, but still included in the analysis set, was 8743 (18.9%) in Cohort 1, 9400 (28.6%) in Cohort 2, and 28,028 (18.6%) in Cohort 3.

All patients received care in the United States; the majority were in New York, California, Florida, and Texas. The five most frequently used payors in this dataset were: Medicare Part B, United Healthcare, Blue Cross/Blue Shield, Humana, and State and Unspecified Medicare. Twenty-six payors served at least 1000 patients each. Another ~4000 payors were associated with fewer than 1000 patients each: there were 713 claims for the AIDS Drug Assistance Program.

Baseline characteristics were determined on the first day of the observation period (Table 1). The median age was 64 for Cohort 1 and 65 for Cohort 2; Cohort 3 was statistically older (median = 69; *p* < 0.001). There were significantly more men in Cohorts 1 (N = 34,226, 74.1%) and 2 (N = 22,407, 68.1%) than in Cohort 3 (N = 57,336, 38.3%); *p* < 0.0001). These differences are consistent with US statistics for HIV infection [17]. Ten NRTIs, five NNRTIs and 11 PIs were prescribed to patients in Cohorts 1 and 2 (Table 2). Notably, Cohort 2 also included 29,808 (90.54%) patients with HIV who did not submit prescription claims for NRTIs, NNRTIs, or PIs yet were alive during the observation period, which may reflect cART exposure through other access mechanisms.

### 2.2. Antiretroviral Exposure and New AD Incidence

**Figure 2:**
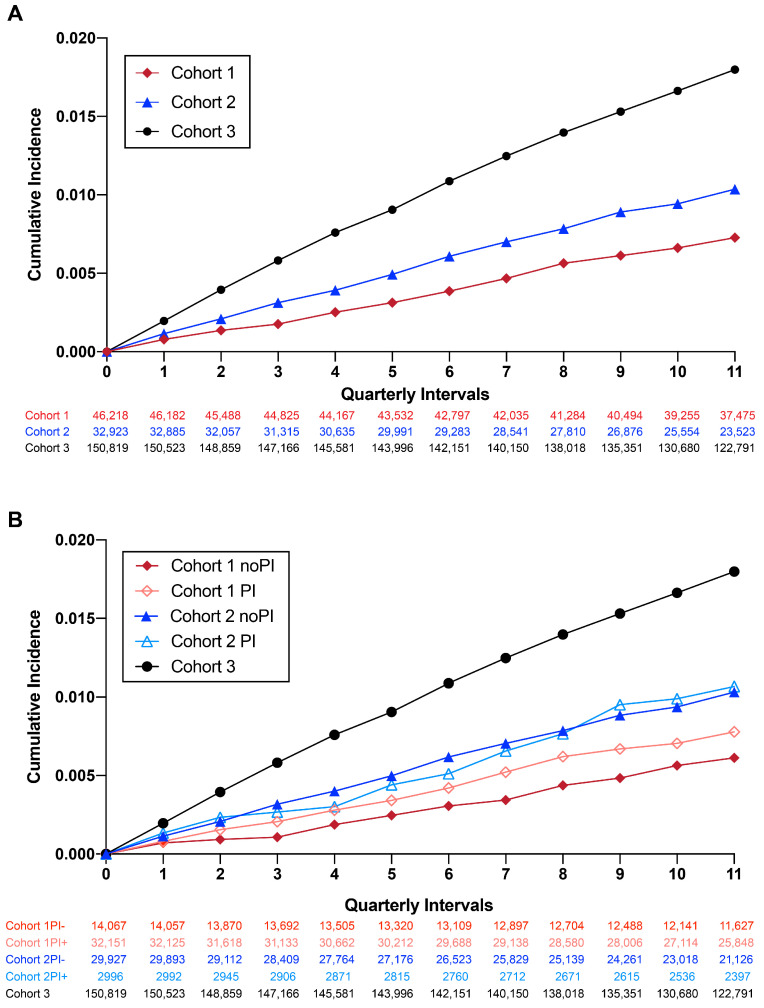
Cumulative incidence of new AD cases during the observation period. (**A**) Cumulative incidence of AD in all three cohorts. (**B**) Cumulative incidence of AD in all three cohorts stratified by PI use.

The cumulative incidence of new AD diagnoses throughout the observation period was calculated on a quarterly basis for each of the three cohorts (Figure 2A) and sub-groups (Figure 2B). We identified statistically significant increases in AD incidence with age and sex, regardless of cohort (Table S1), consistent with known risk factors for AD [1]. Significant differences between cohorts (Table 1) merited age and sex adjustment, which was included in all subsequent analyses. The AD incidence rate per 1000 persons was 2.46 for Cohort 1 (HIV+/NRTI+) 3.55 for Cohort 2 (HIV+/NRTI−) and 6.15 for Cohort 3. A lower incidence was observed in Cohort 1 (HIV+/NRTI+) compared to Cohorts 2 and 3 for all age groups examined (Table S2). A sub-group analysis of Cohorts 1 and 2 identified an increased cumulative AD incidence for patients with PI exposure (Figure 2B). In Cohort 1, patients without PI exposure had the lowest incidence of new AD cases per 1000 person-years (2.06 yrs vs. Cohort 1 with PI = 2.64 yrs; Cohort 2 without PI: 3.55 yrs; Cohort 2 with PI: 3.61 yrs; Cohort 3: 6.15 yrs).

**Figure 3:**
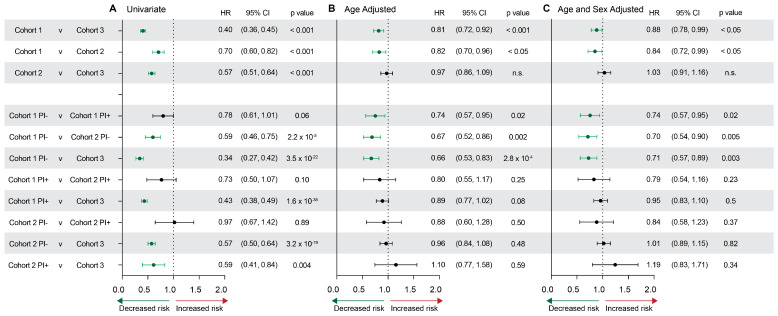
Hazard ratios for AD risk assessed by cohort and stratified by PI use. (**A**) Univariate, (**B**) age-adjusted, and (**C**) age- and sex-adjusted hazard ratios, confidence intervals, and *p* values for each cohort comparison. Green indicates statistically significant hazard ratios that indicate a significantly decreased risk for AD.

Hazard ratios were then used to compare the relative probabilities of developing AD for each cohort (Figure 3). Cohort 1 (HIV+, NRTI+) showed a significantly decreased hazard ratio of 0.84 [0.72, 0.99] when compared with Cohort 2, and 0.88 [0.78, 0.99] when compared with Cohort 3. Notably, Cohort 2 was statistically significant in initial analyses but indistinguishable from Cohort 3 after adjusting for the noted age and sex differences between groups (Figure 3A vs. Figure 3B,C). In all comparisons, Cohort 1 without PI exposure showed a significantly decreased hazard ratio for AD after adjusting for both age and sex (Cohort 1 PI- vs. Cohort 1 PI+: 0.74 [0.57,0.95]; Cohort 1 PI- vs. Cohort 2 PI-: 0.70 [0.54,0.90]; Cohort 1 PI- vs. Cohort 3: 0.71 [0.57, 0.89]) (Figure 3). No differences were observed after age and sex adjustment within Cohort 2 sub-groups or between Cohort 2 and controls.

## 3. Discussion

Patients with HIV who were ≥60 years of age and therefore at risk for AD and who were prescribed NRTIs (Cohort 1; HIV+/NRTI+) had a significantly reduced incidence of new AD compared to patients with HIV who were not prescribed NRTIs (Cohort 2; HIV+/NRTI−) over the 2.75-year observation period. Adjusted hazard ratios were significantly decreased for Cohort 1 patients, with further reductions in those without PI exposure.

Dysregulation of SGR in the AD brain requires RT activity, providing a rationale for RT inhibition as a potential AD treatment. Expectations based on a null hypothesis of no effect contrasted with statistically significant reductions in AD incidence in patients receiving NRTI therapy, suggesting that treatment with NRTIs may prevent or delay AD onset. The effect of NRTIs within the AD brain is most consistent with the inhibition of dysregulated SGR in post-mitotic neurons, considering the proposed mechanism [3] and mid-life initiation of RTIs at an age when neurogenesis has ended. Orthosteric NRTIs (Cohort 1) bind in the active site on HIV and endogenous RTs, and appear to be more effective than allosteric NNRTIs (Cohort 2), which bind to other parts of the HIV RT protein surface and were all developed against heterodimeric HIV RTs. NNRTIs generally lack effect against monomeric RTs from other viruses, and are a major source of predicted monomeric brain RTs (e.g., LINE-1 ORF2 [22,23] or HERV-pol [24]). This is consistent with the hypothesized SGR mechanism that utilizes endogenous human brain RTs [3,4,5] independent of HIV-infection. NRTIs are also known to inhibit inflammasome activation [25,26,27], which has been implicated in the etiology of AD [28], supporting further exploration of current and novel NRTIs and allosteric NNRTIs designed against endogenous RTs for the treatment of AD [28,29,30]. NRTIs may also inhibit RTs present in the brain because of viral infection. Notably, since the completion of this study, multiple groups have identified a significant reduction in cognitive decline in AD-relevant mouse models following NRTI treatment [31,32,33], providing support for our real-world findings.

A further distinction was identified via the negative effects of PIs on AD incidence. cART cocktails often include PIs that are aspartyl protease inhibitors, which could therefore inhibit AD γ- and β-secretases, which are also aspartyl proteases [16,34,35]. Presenilin 1 and 2 mutations are causal for familial AD and encode γ-secretase, which cleaves APP to form Aβ [34]; however, ambiguity over whether these are gain- or loss-of-function AD mutations persists. Our results of PI exposure countering the decreased AD incidence observed with NRTI exposure alone are consistent with loss-of-function secretase mechanisms, as shown in knock-out mice and biochemical studies [35,36,37]. Therefore, the already significant beneficial effects of NRTIs on AD incidence may be improved by using them without concomitant PIs.

Our claims database identified 86,123 patients with HIV who were ≥60 years of age, which represents 50.6% of the 170,108 persons ≥60 years of age living with HIV in the United States in 2016 [17]. Similarly, our study captured 69.4% of the 124,178 people with HIV (≥55 years) receiving some medical care (≥1 CD4 or VL tests) and 84.8% of 101,554 people with HIV (≥55 years) receiving continuous medical care (≥2 CD4 or VL tests) [38]. These statistics demonstrate the completeness of the combined medical claims and prescription claims database capture.

Literature from 15 years ago predicted a significant increase in AD cases among patients with HIV [19,20,39]. However, the first peer-reviewed case of possible AD with co-occurring HIV did not emerge until 2016 [18], supporting a markedly lower incidence of AD in patients treated with cART. Several recent epidemiological studies examining elements of cART on broad neurocognitive endpoints (such as general dementias) reported findings consistent with the present study, albeit in younger patients (median or average age 45–50) [40,41], including those with a short drug exposure (<1 year) [42]. Interestingly, consistent with the detrimental effects of PIs noted here, PIs were also observed to worsen a range of age-related co-morbidities in patients with HIV, including those with dementia [41].

The discrepancy between earlier projections and current AD cases may, in part, be due to neuropathological changes observed in the brains of people with HIV prior to the use of cART, leading to an evolving definition of HIV-associated neurocognitive disorder (HAND). Prior to cART, HAND was uniformly severe and associated with advanced AIDS and premature death [43]. The implementation of cART changed the clinical definition of HAND to a far milder and generally manageable entity, which notably is characterized as reversible and, therefore, fundamentally different from the relentless and progressive nature of AD [44,45,46,47]. At least some biomarkers observed in cognitively impaired patients with HIV are distinct from AD (e.g., Aβ42/Aβ40 ratios are not reduced in HIV, contrasting with AD) [48]. Moreover, neuropathological differences have emerged between HAND and AD, including differences in amyloid plaque morphology, which is typically diffuse in HAND, contrasting with AD, which is characterized by dense-core plaques [49] and where diffuse plaques are considered benign lesions [50,51].

Clinical interpretations from this study are limited by post hoc study design. These limitations include: (1) assessments restricted to patients who use medical care and prescriptions with some regularity; (2) an inability to access drug-use claims filed prior to the exposure period; (3) a brief observation period (2.75 years), which may impact incidence rates; (4) inexact mortality reporting; (5) a reliance upon non-autopsy-confirmed diagnoses by all practitioner types; and (6) imperfectly matched control groups. Limitations related to study design would apply to all cohorts equally.

This post hoc assessment was restricted to patients who use medical care and prescriptions with some regularity. The database is generally representative of the US patient population [52]; however, the HIV-infected cohorts were mostly men (68% and 74%), while the control group was not (38%). Also, the median age of the control cohort was 4 to 5 years higher than the HIV-infected cohorts. Together, these may affect the higher incidence observed in the control cohort as age and sex are two major risk factors for AD, although compensatory adjustments were used to calculate hazard ratios and AD incidence.

Notably, only 10% of Cohort 2 was taking at least one component of cART during the 10-year observation period. However, ~90% of Cohort 2 did not fill prescriptions for NRTIs, NNRTIs, or PIs, which suggests probable use of other forms of cART, including integrase inhibitors, CCR5 antagonists, or fusion inhibitors, which were not assessed in our study, or access to NRTIs and/or related agents through prescription mechanisms not included in this database. It is also possible that patients in Cohort 2 began taking NRTIs during the observation period. Overall, 61% of patients with HIV in our study were taking cART during the exposure period, which is consistent with prior assessments [52,53], including from a recent report in patients >60 years old [54].

We chose to use the “reported alive” method to estimate mortality in the absence of an insurance code or claim for death. This method may result in an overestimation of mortality, but is favorable to the “presumed alive” method that can overestimate survival [21]. Notably, our mortality estimates for Cohorts 1 and 2 combined (21.1%) are congruent with combined mortality rates of people with HIV from 2017 of 12.5% for ages 60 to 64 and 6.7% for ages ≥65 [17], but the mortality rates are high in Cohort 3 (18.6%).

Our analyses were also limited by a relatively low overall number of new AD incident cases, likely a result of the short observation period, in addition to biological effects (e.g., the youngest patients in the cohorts were at very low risk for AD during the observation period). Possible co-morbidities were outside of the scope of this study. The Alzheimer’s Association [1] and Alzheimer’s Disease International [55] estimate that for persons ≥60 years old, 12–18% show mild cognitive impairment (MCI) and ~5–10% have AD (≥65 years, increases with age) [56] with an estimated annual incidence rate of 2.3 to 3.6% (≥65 years) [57]. Our control group AD incidence rates are lower than these previous reports (6.15/1000 reported here vs. 23/1000 [57] for all ages; 21.5/1000 reported here (Table S2) vs. 76/1000 [57] for ages 80–85 and older), which may have resulted in a bias towards the null hypothesis.

Interpretations are also limited by the fact that administrative claims data are not collected to address a specific question and are sub-optimal for incidence-based analyses. For example, this database does not include the date of HIV diagnosis, which limited the analyses that could be completed. The assessed claims were open (not adjudicated by a payer/insurer) and knowingly incomplete for outpatient hospitalization claims. The prescription information was reasonably complete. Future analyses of completed and adjudicated claims should also be pursued.

## 4. Materials and Methods

### 4.1. Data Sources, Study Population and Selection of Cohorts

This retrospective, observational, United-States-population-based cohort study evaluated administrative claims data assembled by IQVIA Inc. This proprietary data set links non-adjudicated medical claims to longitudinal prescription claims using anonymous patient tokens, permitting longitudinal linkage of patient histories. The medical claims dataset captured ~75% of American Medical Association providers, and the prescription claims dataset captured approximately 85% of all pharmacy benefit prescriptions in the US, including Medicaid. Individuals were de-identified at the source of data entry and integrated into a database that contains limited patient demographics, health insurance payors, and medical and pharmacy claims from over 200 million unique patients. The IQVIA anonymization process is certified as compliant with the Health Insurance Portability and Accountability Act and exempt from Institutional Review Boards, and therefore the study did not require individual consent to participate.

Data from 1 January 2005 to 30 June 2019 (study design in Figure 1A) were analyzed in September–December 2019. The exposure period extended from 1 January 2005 to 30 September 2015, the selection period extended from 1 October 2015 to 30 September 2016, and the selection and observation periods were selected for the exclusive use of the International Statistical Classification of Diseases and Health Related Problems revision 10 (ICD-10) codes and occurred between 1 October 2016 and 30 June 2019 (2.75 years). ICD-10 use was mandated by the first day of our selection period (1 October 2015) and its sole use provided consistency between the exposure, selection, and observation periods.

Patients who placed a medical billing claim during the selection period and who met the eligibility criteria were partitioned into three cohorts. Cohorts 1 and 2 contained patients with an HIV diagnosis (ICD-10 codes B20 and Z21) who were aged ≥60 years and placed medical billing claims during the selection period. Patients that were prescribed at least one NRTI during the exposure period were included in Cohort 1 (HIV+/NRTI+) and patients that were not prescribed NRTIs during the exposure period were included in Cohort 2 (HIV+/NRTI™). Using assigned HIV infection ICD codes has been validated as sensitive (96.2%, 95% CI 95.2–97.9%) and specific (99.6%, 95% CI 99.1–99.8%) [58]. Patients with an existing AD diagnosis (ICD-10 codes G30, G30.0, G30.1, G30.8, or G30.9) prior to the observation period (i.e., during the exposure or selection period) or without claims filed during the observation period were excluded. Cohorts 1 and 2 were also sub-grouped according to PI exposure.

Patients diagnosed with common cold or nasopharyngitis (ICD-10 code J00) and who did not have an HIV-infection diagnosis from insurance claims during the selection period were included in Cohort 3 (HIV−/NRTI−). We chose common cold as a benign but frequent diagnosis that would indicate the use of medical treatment and an identical path for claims data. Exclusion criteria for this cohort included the use of any NRTIs, NNRTIs, or PIs during the exposure and selection periods or a hepatitis B diagnosis (ICD-10 code B16*). There was no sub-grouping for Cohort 3 because PI exposure was part of the exclusion criteria. Patients with missing data required to determine cohort or incidence rates were excluded.

### 4.2. Statistical Analyses

Baseline characteristics for each cohort were determined on the first day of the observation period. The primary objective was to determine the cumulative incidence of AD that developed during the 2.75-year observation period for comparison between the three cohorts. The secondary objective was to determine the cumulative incidence of AD that developed during the 2.75-year observation period by sub-grouping Cohorts 1 and 2 according to PI exposure. The cumulative incidence was calculated as the number of AD cases divided by the total number of individuals in the at-risk population at quarterly time intervals. The index date/study start date was the first day of the observation period (1 October 2016) when all groups were considered equal with no individuals having AD. Mortality was approximated by the “reported alive” method for calculating survival by the absence of insurance claims placed over the observation period by using informed censoring of subjects at the end of the last quarter where a claim was filed [21]. This method has been described as the most conservative way to estimate outcomes without a specific medical claim diagnosis or procedure code to confirm death directly. Other reasons for not placing a medical or prescription claim include patient dis-enrollment from a payor and/or an enrollment change to a provider or a pharmaceutical distributor not included in the database.

Hazard ratios were used to compare the relative probabilities of developing AD. Hazard ratios and confidence intervals (CIs) were estimated with Cox Proportional Hazard modeling, adjusting for the covariates specified. The proportionality assumptions across cohorts over time were confirmed visually with negative log survival curves estimated with the Kaplan–Meier method. All statistical estimations were carried out in SAS 9.4. For the primary objective of testing between cohorts (Cohort 1 vs. 2; Cohort 1 vs. 3; and Cohort 2 vs. 3), we performed sequential comparisons to control the overall Type 1 error. The test order was (1) test the difference in cumulative incidence of AD for Cohort 1 vs. 2; if (1) is statistically significant, then test (2) the difference in cumulative incidence for Cohort 1 vs. 3 and (3) Cohort 2 vs. 3. The secondary objective was exploratory, and no multiplicity adjustments were made; the *p* value to signify statistical significance was *p* < 0.05. Exact *p* values are provided where possible.

## 5. Conclusions

We identified a statistically significant positive association between NRTI exposure and decreased risk for sporadic AD in patients with HIV and ≥60 years of age. Patients receiving NNRTIs also showed a decreased risk, which could reflect non-prescription exposure to NRTIs and/or beneficial effects of other cART constituents. AD risk reduction was countered in all examined conditions by PI exposure, consistent with possible loss-of-function effects on γ and/or β-secretase—both of which are aspartyl proteases that may be inhibited by PIs—and in concert with the scientific literature linking familial mutations in presenilins to their loss of function in AD [35,37]. Post-marketing surveillance of NRTIs has shown acceptable safety data sufficient to allow NRTIs to be prescribed, as a class, continuously since 1987, and tens of thousands of patients ≥60 years of age are currently taking these medications, providing support that these agents will be well tolerated in aged patients. The data presented here support controlled clinical trials using NRTIs on patients with mild cognitive impairment (MCI), pre-symptomatic familial AD, Down syndrome, and sporadic AD, along with asymptomatic APOE4 carriers. Research on the products of SGR that may identify mechanisms, biomarkers [8] or endogenous brain source(s) of RTs, which are undoubtedly distinct from HIV RT, should also be pursued.

## Figures and Tables

**Table 1:** Baseline demographics by cohort.

	Cohort 1 HIV+/NRTI+ N = 46,218	Cohort 2 HIV+/NRTI− N = 32,923	Cohort 3 HIV−/NRTI− N = 150,819
**Median Age, yrs ^a^**	64	65	69 ^b^
**Men, N (%) ^c^**	34,226 (74.1%)	22,407 (68.1%)	57,336 ^d^ (38.3%)
**History of IV drug-use, N (%)**	1990 (4.3%)	1221 (3.7%)	708 (0.47%)
**Hemophilia, N (%)**	105 (0.2%)	44 (0.1%)	84 (0.06%)

^a^ The age range for all groups was 60–86; after age 86 the system only reports 86+. ^b^ Kruskal–Wallis test with 1 degree of freedom, H = 113,550 (Cohort 1 vs. 3), *p* < 0.0001; H = 6817 (Cohort 2 vs. 3), *p* <0.0001. ^c^ Sex was not specified for 15 patients; they were excluded from all sex-adjusted analyses. ^d^ Chi-squared Test with 1 degree of freedom, Χ^2^ = 18,468 (Cohort 1 vs. 3), *p* < 0.0001; Χ^2^ = 9944 (Cohort 2 vs. 3), *p* < 0.0001.

**Table 2:** cART prescription claims for Cohort 1 and Cohort 2 patients.

	Cohort 1 HIV+/NRTI+ N = 48,571	Cohort 2 HIV+/NRTI− N = 37,252
**Nucleoside Reverse Transcriptase Inhibitors (NRTIs)** **N (%, median duration in yrs)**		
emtricitabine/tenofovir disoproxil fumarate	28,455 (61.6%, 4)	0 (0.0%, 0)
abacavir sulfate/lamivudine	13,256 (28.7%, 4)	0 (0.0%, 0)
tenofovir disoproxil fumarate	12,281 (26.6%, 3)	0 (0.0%, 0)
lamivudine	10,901 (23.6%, 2)	0 (0.0%, 0)
abacavir	7134 (15.4%, 3)	0 (0.0%, 0)
didanosine	4392 (9.5%, 3)	0 (0.0%, 0)
Abacavir sulfate/lamivudine/zidovudine	4085 (8.8%, 3)	0 (0.0%, 0)
stavudine	3754 (8.1%, 2)	0 (0.0%, 0)
zidovudine	2689 (5.8%, 2)	0 (0.0%, 0)
emtricitabine	2693 (5.8%, 2)	0 (0.0%, 0)
**Non-Nucleoside Reverse Transcriptase Inhibitors (NNRTIs)** **N (%, median duration (yrs))**		
efavirenz	11,239 (24.3%, 3)	1997 (6.1%, 4)
nevirapine	5857 (12.7%, 5)	703 (2.1%, 5)
etravirine	4774 (10.3%, 4)	443 (1.4%, 3)
rilpivirine	1292 (2.8%, 2)	64 (0.2%, 2)
delavirdine	223 (0.5%, 3)	15 (0.1%, 6)
**Protease Inhibitors (PIs)** **N (%, median duration (yrs))**		
ritonavir	23,844 (51.6%, 4)	1362 (4.1%, 3)
atazanavir	14,808 (32.0%, 4)	602 (1.8%, 4)
darunavir	12,111 (26.2%, 4)	786 (2.4%, 3)
lopinavir/ritonavir	10,283 (22.3%, 3)	816 (2.5%, 4)
fosamprenavir	4362 (9.4%, 3)	169 (0.5%, 4)
nelfinavir	2563 (5.6%, 3)	490 (1.5%, 5)
darunavir/cobicistat	2067 (4.5%, 1)	165 (0.5%, 1)
indinavir	1072 (2.3%, 2)	290 (0.9%, 4)
saquinavir	1392 (3.0%, 3)	153 (0.5%, 4)
tipranavir	641 (1.4%, 2)	12 (0.04%, 2)
atazanavir/cobicistat	468 (1.0%, 1)	18 (0.1%, 4)

## Data Availability

All datasets generated and analyzed in this study are owned by IQVIA and are not publicly available. These data sets may be requested through IQVIA.

## Supplementary Materials

The following supporting information can be downloaded at: https://www.mdpi.com/article/10.3390/ph17040408/s1, Table S1: Hazard ratios for AD risk as a factor of age and sex, regardless of cohort; Table S2. Incident AD cases sub-grouped by age.
