# Supplementary material for: Nucleoside Reverse Transcriptase Inhibitor Exposure Is Associated with Lower Alzheimer’s Disease Risk: A Retrospective Cohort Proof-of-Concept Study
Source: Pharmaceuticals (Basel). 2024 Mar 22;17(4):408. doi: 10.3390/ph17040408 (PMC11053431; doi:10.3390/ph17040408)
Supplement: Supplementary file 1 [file pharmaceuticals-17-00408-s001.zip › pharmaceuticals-2893219-supplementary.pdf]

Nucleoside Reverse Transcriptase Inhibitor Exposure is Associated with Lower Alzheimer's Disease Risk: A Retrospective Cohort Proof-of-Concept Study

Chow *et al.*

Table of Contents

Supplemental Tables

Page 2: Table S1: Hazard ratios for AD risk as a factor of age and sex, regardless of Cohort (Cohorts were combined for analysis).

Page 3: Table S2: Incident AD cases sub-grouped by age.

Table S1. Hazard ratios for AD risk as a factor of age and sex, regardless of Cohort.

	Age-Adjusted	Age & Sex-Adjusted
**Cohort 1 (HIV+/NRTI+) & Cohort 3 (HIV-/NRTI-)**		
70-79 years of age	4.33	4.34
80+ years of age	12.98	12.91
Female		1.27
**Cohort 1 (HIV+/NRTI+) & Cohort 2 (HIV+/NRTI-)**		
70-79 years of age	4.33	4.31
80+ years of age	10.56	9.89
Female		1.61
**Cohort 2 (HIV+/NRTI-) & Cohort 3 (HIV+/NRTI-)**		
70-79 years of age	4.30	4.31
80+ years of age	13.12	13.05
Female		1.27
Cohorts were combined for analyses. The reference group was 60-69 years of age; $p < .001$ for all values.		

Table S2. Incident AD cases sub-grouped by age.

Age Group (yrs)	N (% of Cohort)	Number of Incident AD Cases	Incidence rate per 1000 person-years
**Cohort 1 (HIV+/NRTI+)**			
60-69	37,224 (80.5%)	143	1.37
70-79	7619 (16.5%)	129	6.21
80+	1375 (3.0%)	44	12.29
**Cohort 2 (HIV+/NRTI-)**			
60-69	25,003 (75.9%)	111	1.65
70-79	5981 (18.1%)	108	6.85
80+	1939 (5.9%)	93	19.45
**Cohort 3 (HIV-/NRTI-)**			
60-69	81,652 (54.1%)	370	1.58
70-79	44,739 (29.7%	872	6.89
80+	24,428 (16.2%)	1365	21.50
